# Insufficient evidence for the use of a physical examination to detect maltreatment in children without prior suspicion: a systematic review

**DOI:** 10.1186/2046-4053-2-109

**Published:** 2013-12-06

**Authors:** Eva MM Hoytema van Konijnenburg, Arianne H Teeuw, Tessa Sieswerda-Hoogendoorn, Arnold G E Leenders, Johanna H van der Lee

**Affiliations:** 1 Department of Paediatrics, Emma Children’s Hospital/Academic Medical Centre, University of Amsterdam, Postbus 22660, Amsterdam, 1100 DD, The Netherlands; 2 Medical Library, Academic Medical Centre, University of Amsterdam, Postbus 22660, Amsterdam 1100 DD, The Netherlands; 3 Academic Medical Centre Amsterdam, Department of Paediatrics, University of Amsterdam, Room K01-138, Meibergdreef 9, Amsterdam 1105 AZ, The Netherlands

**Keywords:** Child abuse, Diagnosis, Physical examination

## Abstract

**Background:**

Although it is often performed in clinical practice, the diagnostic value of a screening physical examination to detect maltreatment in children without prior suspicion has not been reviewed. This article aims to evaluate the diagnostic value of a complete physical examination as a screening instrument to detect maltreatment in children without prior suspicion.

**Methods:**

We systematically searched the databases of MEDLINE, EMBASE, PsychINFO, CINAHL, and ERIC, using a sensitive search strategy. Studies that i) presented medical findings of a complete physical examination for screening purposes in children 0–18 years, ii) specifically recorded the presence or absence of signs of child maltreatment, and iii) recorded child maltreatment confirmed by a reference standard, were included. Two reviewers independently performed study selection, data extraction, and quality appraisal using the QUADAS-2 tool.

**Results:**

The search yielded 4,499 titles, of which three studies met the eligibility criteria. The prevalence of confirmed signs of maltreatment during screening physical examination varied between 0.8% and 13.5%. The designs of the studies were inadequate to assess the diagnostic accuracy of a screening physical examination for child maltreatment.

**Conclusions:**

Because of the lack of informative studies, we could not draw conclusions about the diagnostic value of a screening physical examination in children without prior suspicion of child maltreatment.

## Background

Child maltreatment is a worldwide problem with many adverse consequences, both in the short and long term [1-5]. Early detection of child maltreatment is extremely important in order to intervene and improve the situation, and to prevent recurrence, severe morbidity, or even death [6-9]. The large discrepancy between the much higher prevalence of self-reported maltreatment compared to the prevalence of maltreatment of which professionals are aware, even when using identical criteria, means that a substantial amount remains undetected [1,8-10]. The contribution of hospitals to the total number of child maltreatment reports is relatively small. Several studies have shown that child maltreatment is under-detected by hospital staff [9,11,12]. To improve the detection of child maltreatment in hospitals, a number of strategies, such as checklists and training of personnel, have been developed [13,14]. Another strategy that is widely used in emergency departments and other health care settings to detect child maltreatment is to perform a screening physical examination. The physical examination is targeted towards exposing signs of child maltreatment, and is sometimes called ‘top-to-toe’ inspection. In the Netherlands, 41% of Dutch emergency departments use a physical examination as a screening tool, mainly in younger children [15]. The examination can also be used as part of a broader screening tool, for example, as part of a checklist [13,16-19]. In these settings, the physical examination is used as a screening tool in all children (without prior suspicion of maltreatment), and thus performed regardless of the complaints of the child.

A screening physical examination is relatively easy, inexpensive, and in principle without adverse effects. During the examination, the child is undressed completely and specifically inspected for any signs of physical abuse and physical neglect (e.g., scars, bruises, caries, unkempt appearance). Furthermore, abnormal physical and emotional development, behaviour and parent–child interaction can be observed. All of the above could lead to suspicions of child maltreatment. Depending on the age of the child, the physical examination is likely to show different findings according to the child’s physical development and the mechanism of abuse (for example, abusive head trauma is usually seen in very young children, presenting with specific features) [20]. The physical examination might be most relevant in young, non-verbal children, who are unable to talk about maltreatment. Possible undesirable effects of a screening physical examination might occur if a negative screening result is falsely reassuring for professionals or if the result is a false positive. In addition, it could be that maltreating parents are discouraged from visiting a health care setting if they know that their children will be physically examined for possible maltreatment. A screening physical examination would mostly identify physical abuse and neglect, and can never identify all forms of child maltreatment. Therefore, it is generally used in combination with other screening strategies in order to increase the sensitivity of child maltreatment detection [21].

To our knowledge, although many child maltreatment protocols in various health care settings include a screening physical examination and clinicians rely on the results, the diagnostic value of a screening physical examination to detect maltreatment in children without prior suspicion has not yet been reviewed. Two systematic reviews investigated the performance of various screening methods for maltreatment in children presenting at emergency departments [14,22]. Of all 17 studies included in both reviews, only one study investigated a complete physical examination as part of a screening method, in combination with a checklist and discussion with a physician [23]. However, the diagnostic value of this physical examination is unclear since results were not reported separately from the other aspects of the screening method. Evidence suggestive of abuse was found in 10% [24] and 63% [25] of children who were physically examined because of (suspected) maltreatment. However, the physical examination probably yields different results when used as a screening method to detect child maltreatment in children without prior suspicion. Although a screening physical examination is often (but not always) performed in combination with other screening tools, it is important to also assess its added diagnostic value. In practice, clinicians use the results of the screening physical examination to make a risk assessment, and should therefore know its diagnostic value. If physicians are unaware of this, they might over- or under-detect child maltreatment, which can have serious adverse consequences.

We therefore performed a systematic review to evaluate the diagnostic value of a complete physical examination, minimally consisting of a visual inspection of the entire skin and oral cavity, as a screening instrument for maltreatment in children without prior suspicion in various health care settings compared to a ‘composite reference standard’ (a combination of reference standards, considered to be positive if at least one of the components is positive). Unfortunately, no gold standard is available for child maltreatment; therefore, to determine diagnostic test accuracies, derived standards have to be used as a reference, i.e., a diagnosis of maltreatment by either i) a court, ii) the Child Protective Services (CPS), iii) an expert panel, iv) a forensic physician, or v) self-report.

## Methods

### Search methods

We systematically searched the electronic databases of MEDLINE (through PubMed and through Ovid, from 1947 to August 8, 2013), EMBASE (1980 to August 8, 2013), PsychINFO (1806 to August 8, 2013), CINAHL (1982 to August 8, 2013) and ERIC (1965 to August 8, 2013). The main search strategy consisted of three components combined by ‘AND’, namely ‘physical examination’, ‘child’, and ‘abuse’. Synonyms for these terms were combined with the corresponding component with ‘OR’. Furthermore, database-specific MeSH and thesaurus terms and text words were added. Because we expected a small number of eligible articles, we used a sensitive search strategy. See Additional file 1 for full search strategies.

The lists of cited and citing references of included articles and of articles that were considered for inclusion at an early stage were hand searched for additional relevant articles. Furthermore, 13 key authors were approached and asked if they could recommend any relevant studies in this area. Finally, articles known by any of the authors were added to the search results. We searched for both published and unpublished reports. There was no language restriction.

### Study selection

The optimal study design to answer our research question would be a cross-sectional diagnostic accuracy study, comparing a physical examination to a universal reference standard in all children. However, because we did not expect to find many studies, we broadened our eligibility criteria to the following: any empirical study including children 0–18 years visiting any health care setting, evaluating a complete physical examination (defined as minimally consisting of a visual inspection of the entire skin and oral cavity) specifically performed as (part of) a screening procedure for child maltreatment by a health care professional and providing systematic documentation of the presence or absence of signs indicating child maltreatment in comparison to one of the following reference standards: i) a court, ii) the CPS, iii) an expert panel, iv) a forensic physician, or v) a self-report. Since the aim of this review was to evaluate the physical examination as a diagnostic instrument to detect maltreatment in children without prior suspicion, we did not include studies in children who were physically examined because of (or a suspicion of) child maltreatment. Furthermore, because this review aims to evaluate the complete ‘top-to-toe’ examination, studies were excluded if the physical examination was only performed with the intention to detect sexual child abuse.

Two reviewers (EH, AT) selected the studies independently, first based on titles, then on abstracts and keywords, and finally on full texts. Disagreements were discussed until consensus was reached.

### Data extraction and assessment

Appraisal of the methodological quality and data extraction were performed by both reviewers (EH, AT) independently. Data from included studies were extracted and the methodological quality was assessed with a combined form including data extraction items and the items of the QUADAS-2 tool for quality assessment [26]. The combined form was piloted independently and adjusted by two reviewers until there was consensus on a final version. Disagreements in data extraction or quality assessment were discussed until consensus was reached. Where necessary, a third reviewer (TS) was the final judge. Extracted data included: i) characteristics of the study (design, year of publication, type of publication, study country, funding source); ii) characteristics of the study population (including age, sex distribution, previous diagnosis of child maltreatment); iii) characteristics of the screening physical examination (including setting); iv) characteristics of the reference standard; v) characteristics of the outcome measure (child maltreatment diagnosis, type of maltreatment findings); vi) any reported harm caused by a screening physical examination; vii) sensitivity (true positives, proportion of maltreated children with a positive physical examination), and viii) specificity (true negatives, proportion of non-maltreated children with a negative physical examination).

## Results

**Figure 1:**
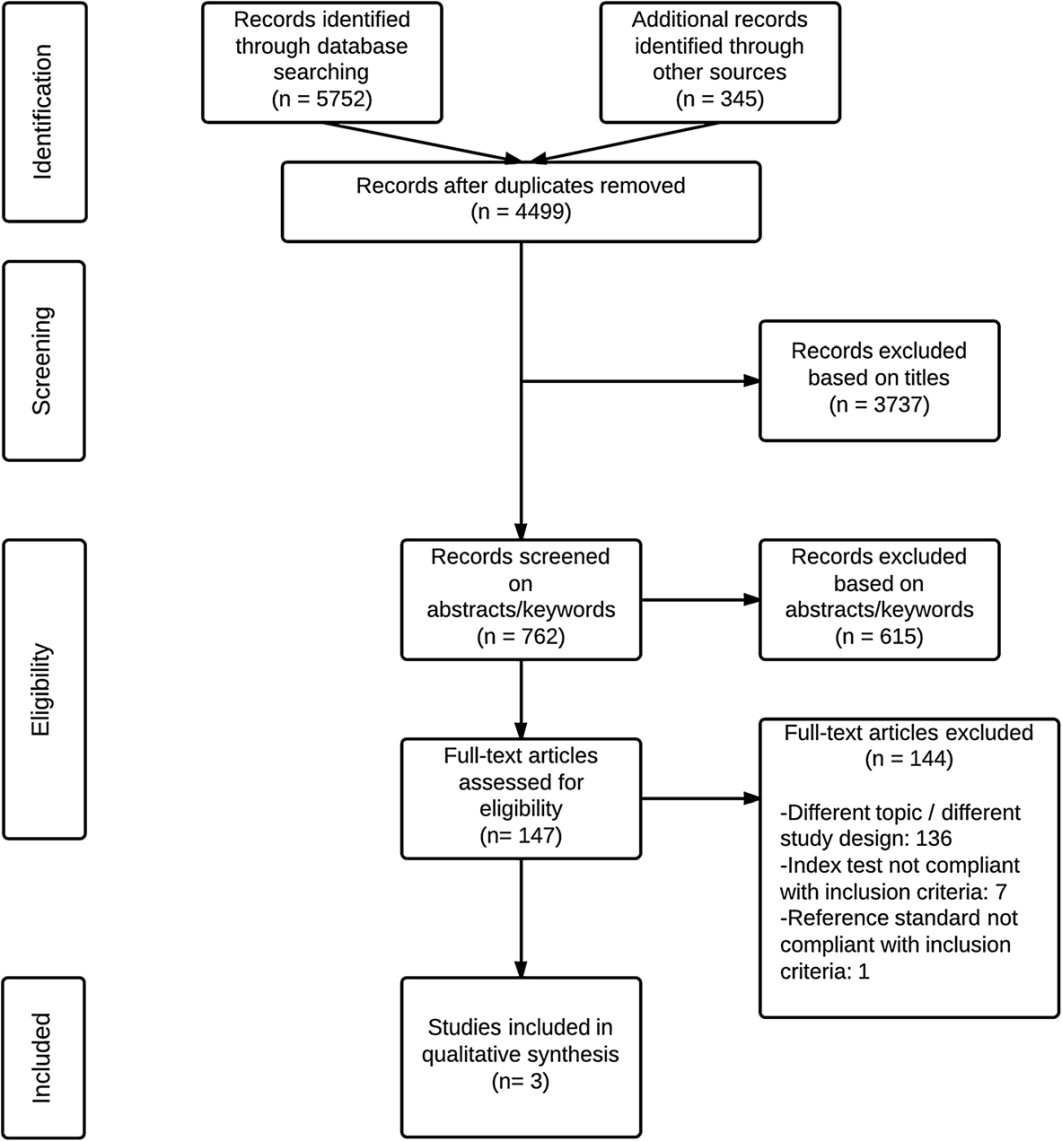
PRISMA flow diagram.

The electronic literature search provided 4,215 titles after the removal of duplicates. Furthermore, 284 studies, retrieved by citation search, personal knowledge or communication with key authors, were added after the removal of duplicates. Of all these, 762 studies were selected based on title and subsequently 147 studies were selected based on abstract and keywords of which full text studies were read. Application of inclusion and exclusion criteria led to the inclusion of three studies [27-29]. Figure 1 presents the study selection process with reasons for exclusion in a PRISMA flow diagram [30].

The characteristics of the three included studies are presented in Table 1. None of the included studies were designed to evaluate the diagnostic accuracy of the physical examination to identify child maltreatment. Two studies were designed to determine the prevalence and associated risk factors for child maltreatment, one study in a community sample [27] and one study in children visiting the emergency department [28]. The third study was designed to evaluate a screening tool for child maltreatment, including a complete physical examination, at the emergency department [29]. The reference standards were a child maltreatment diagnosis by the CPS [29], an expert panel [28], and self-report by the child [27].

**Table 1:** Characteristics of included studies

**First author**	**Year of publication**	**Country**	**Type of study**	**Setting**	**Study aim**	**Sample size**	**Age**	**Sex distribution (%) male/female**	**Index test**	**Reference standard**
Afifi [27]	2003	Egypt	Cross-sectional	Preparatory and secondary school students from a rural community, selected by random cluster sampling	To identify the prevalence and underlying risk factors of child maltreatment	555	12–18 years; mean age 15.6 ±1.5 years	63/37	General physical examination by physician, specifically including signs of previous or recent physical abuse	Self-report of the child in combination with positive signs upon physical examination
Palazzi [28]	2005	Italy	Cross-sectional	All children 0–14 years presenting in 19 emergency departments	To identify the prevalence and associated risk factors of suspected child maltreatment	10,175	0–14 years; mean age 4.8 ±3.9 years	57/43	Complete physical examination whenever possible, especially in younger children	Six-point suspicion index for child maltreatment attributed by an expert panel of a local child health team in collaboration with research assistants, based on routine assessments
Rosenberg [29]	1982	USA	Prospective, 1-year follow-up	A randomly enrolled sample of children 0–2 years visiting an emergency department	To prospectively evaluate a brief screening assessment for child maltreatment	476	0–2 years	55/45	Caregiver undresses child, assessment by nurse for being unkempt, having a bald occiput, and the presence of physical bruises, burns or bites	Registered as maltreated at the CPS^i^ (the Department of Social Services) at 1-year follow-up

^i^CPS = Child Protective Services.

### Quality of the studies

The quality of the three studies that were included, according to the QUADAS-2 tool [26], is presented in Table 2. None of the studies contained a flow diagram, thus, a flow diagram was hand-drawn and reviewed for each study.

**Table 2:** **Quality assessment of reviewed studies with QUADAS-2 tool**[26]

	**Patient selection**		**Index test**		**Reference standard**		**Flow and timing**	**Reviewer comments**
	**Risk of bias**	**Concerns about applicability**	**Risk of bias**	**Concerns about applicability**	**Risk of bias**	**Concerns about applicability**	**Risk of bias**	
Affifi (2003) [27]	Low	Low	Low	Low	High	High	Low	Sensitivity and specificity cannot be calculated due to reference standard related bias (reference standard is incorrect due to the use of self-report in combination with signs upon physical examination, which is likely to underestimate true prevalence)
Palazzi (2005) [28]	High	Low	Low	Unclear	High	Low	High	Sensitivity and specificity cannot be calculated, due to i) reporting bias (cumulative prevalence of at least 1 positive finding upon physical examination not being reported) and ii) incorporation bias (results of physical examination are used in establishing the reference standard)
Rosenberg (1982) [29]	Low	Low	Low	Unclear	High	Low	High	Sensitivity and specificity cannot be calculated, due to i) different timing of application of the reference standard, ii) reporting bias (cumulative prevalence of at least 1 positive finding upon physical examination not being reported) and iii) information bias (due to a different definition of physical signs of maltreatment used at the time)

The first study, that of Afifi et al., is a cross-sectional study with a sample size of 555 subjects [27]. The study aimed to identify the prevalence and underlying risk factors of child maltreatment in school-aged children in rural Egypt. This is the only study performed in a community sample. The strengths of the study are its large sample size, the random selection of participants and the participation of all eligible subjects. An important limitation is that subjects were considered non-abused if there were no signs of physical abuse on examination, even when abuse was self-reported. On the other hand, if subjects denied abuse, they were considered non-abused even when positive signs of abuse were present during examination [31,32]. This lead to reference standard related bias, and probably to false negative test results.

Two studies were performed in an emergency department [28,29]. The study by Palazzi et al. is a cross-sectional study with a sample size of 10,175 subjects [28]. The study aimed to identify the prevalence and associated risk factors of suspected child maltreatment in paediatric emergency departments in Italy. The strengths of the study are the very large sample size and the multicentre design. An important limitation is that the results of different parts of the physical examination are presented separately (skin lesions, oral lesions, etc.) and the cumulative prevalence of any positive sign of child maltreatment is unknown, but most likely higher than the reported prevalence of skin lesions only (reporting bias). Furthermore, the results of the physical examination were used to establish the reference standard (incorporation bias).

The third study, that by Rosenberg et al., is a prospective study with a follow-up of one year, including 476 subjects [29]. The study aimed to prospectively evaluate a brief screening assessment for child maltreatment at an emergency department. The strengths of the study are the large sample size, the random enrolment of children (although the randomization process is not described), and the independence of the reference standard for the results of the physical examination. Limitations are that the reference standard was applied at 1-year follow-up and was considered positive if maltreatment had ever occurred. Therefore, it is possible that maltreatment was confirmed, even if this happened after the emergency department visit. Other important limitations, probably leading to underestimation of the prevalence of signs of child maltreatment during physical examination, are that children were excluded from the study if there was a suspicion of child maltreatment before or during the visit to the emergency department, and that the number of children with any positive sign of child maltreatment is not reported. Finally, this study was published in 1982, at which time some views on child maltreatment were different from today (such as considering a bald occiput a sign of maltreatment), possibly leading to information bias.

### Results of the physical examination

Table 3 shows the prevalence of any signs of child maltreatment found upon screening physical examination (unconfirmed) and signs of maltreatment found in children who were indeed maltreated as confirmed by a reference standard (confirmed) as reported in the three included studies. The prevalence of unconfirmed signs of maltreatment ranged between 7.8% and 14.6% of the children examined. The prevalence of signs of child maltreatment confirmed by a reference standard ranged between 0.8% and 13.5%. Due to the study designs, it was impossible to use sensitivity and specificity of the studies to determine the diagnostic accuracy of the screening physical examination to detect child maltreatment. See Additional file 2 for the 2 x 2 contingency tables of the results of the included studies.

**Table 3:** Summary of results of included studies

**Author (year)**	**Children with unconfirmed signs of maltreatment upon physical examination/children examined**	**Children with signs of maltreatment upon physical examination confirmed by reference standard/children examined**
Afifi (2003) [27]	81/555 (14.6%)	75/555 (13.5%)
	(burns 30, bruises 20, scars 19, scratches 10, bite marks 2)	
Palazzi (2005) [28]	Skin lesions: 1,177/9,510 (12.4%)	Skin lesions: 75/9,510 (0.8%)
	Oral lesions: 123/9,137 (1.3%)	Oral lesions: 8/9,137 (0.09%)
	Present or past burns, fractures and head trauma are presented separately in the original article. However, it is unclear whether this is assessed during physical examination and, therefore, these results are not included in this review.	
	The number of children with at least one finding upon physical examination is unknown	
Rosenberg (1982) [29]	Unkempt: 37/473 (7.8%)	Unkempt: 7/473 (1.5%)
	Bruises, burns, human bites: 18/473 (3.8%)	Bruises, burns, human bites: 5/473 (1.1%)
	Bald occiput*: 14/474 (3%)	Bald occiput*: 0/474 (0%)
	The number of children with at least one finding upon physical examination is unknown	

*This is no longer considered a sign of child abuse.

## Discussion

### Main findings

This review did not establish the diagnostic accuracy of a complete physical examination as a screening instrument for maltreatment in children without prior suspicion in health care settings. No studies providing an adequate estimation of sensitivity and specificity of the screening physical examination for child maltreatment could be identified. Three studies were included [27-29]. In these studies, the prevalence of confirmed signs of maltreatment upon a screening physical examination ranged between 0.8% and 13.5%. The risk of bias of the reference standard was considered high for all three studies. The reference standard was not independent of the results of the screening physical examination in two of the three reviewed studies [27,28]. In two studies, results of various aspects of the physical examination were presented separately, and cumulative numbers of children with at least one positive finding upon physical examination were unclear [28,29].

### Strengths and limitations of this review

The strengths of this review are the systematic approach and the extensive literature search. Three studies were included and systematically assessed for methodological quality using the thoroughly developed QUADAS-2 tool. None of these studies investigated any potential harm caused by a physical examination. It was not possible to determine in which subgroups (for example age groups) a screening physical examination was more or less accurate. Unfortunately, because the search did not identify studies that provided sensitivity and specificity, we could not draw conclusions about the diagnostic value of a screening physical examination in children without prior suspicion of child maltreatment.

### Recommendations

Although it is widely used in clinical practice, there is insufficient evidence for a physical examination as a screening instrument to improve detection of maltreatment in children without prior suspicion. Currently, when using a screening physical examination to detect child maltreatment in practice, clinicians should be aware that its diagnostic accuracy is unclear and child maltreatment can be both over- and under-detected. A negative result does not exclude abuse, because i) not all abuse leaves injuries, ii) even serious injuries caused by physical abuse (such as fractures) can be present without any signs upon physical examination, and iii) prior injuries of abuse may already have disappeared [16,31,32]. On the other hand, some findings upon physical examination can mimic physical abuse while being of a non-abusive nature (for example a Mongolian spot) [33,34]. Finally, although it is possible that a screening physical examination could identify emotional or sexual maltreatment in rare cases (for example because of a disclosure during the examination or because abnormal development, behaviour, or parent–child interactions observed), the examination is aimed towards the detection of physical abuse or neglect, and other forms of child maltreatment could be overlooked.

To determine the diagnostic accuracy of a physical examination as a screening instrument to detect maltreatment in children without prior suspicion, we would recommend a study with a protocolized systematic physical examination and reference standard for a large, unselected group of children, at different levels of risk for maltreatment, and in different settings. Although we acknowledge that it is challenging to find an optimal reference standard, it could be a thorough case review by an expert panel in combination with child-, parent-, informant-, and (if there is involvement) CPS-reports. If parents and children are interviewed or asked to fill out a questionnaire in a respectful way, the research would not be a major burden for them, and it would not be unethical to also apply the reference standard to children with a negative screening result. Ideally, to determine the sensitivity, specificity, and positive predictive value of the screening physical examination, all children should undergo the physical examination and the reference standard, regardless of the results of the physical examination. However, in practice, this might not be feasible given the large number of children that would require the (time-consuming) reference test. To solve this issue, the reference standard could be performed in all children with a positive physical examination and in a random sample of children with a negative physical examination, as is currently being done in a study focused on the emergency department [17].

## Conclusions

Because of the lack of informative studies, we could not draw conclusions about the diagnostic value of a screening physical examination in children without prior suspicion of child maltreatment.

## Abbreviations

CPS: Child protective services.

## Competing interests

The authors declare that they have no competing interests.

## Authors’ contributions

EH and AT carried out data extraction, quality appraisal and data analysis, participated in study design, and drafted the manuscript. AL designed the search strategies and revised the manuscript. TS participated in study design and assisted in data extraction and quality appraisal, and revised the manuscript. JL participated in study design, advised on all methodological issues, and helped to draft and revise the manuscript. All authors read and approved the final manuscript.

## Supplementary Material

Additional file 1 Full search strategies. Click here for file

Additional file 2 2 x 2 contingency tables of included studies. Click here for file
