# Supplementary material for: Insufficient evidence for the use of a physical examination to detect maltreatment in children without prior suspicion: a systematic review
Source: Syst Rev. 2013 Dec 6;2:109. doi: 10.1186/2046-4053-2-109 (PMC4029283; doi:10.1186/2046-4053-2-109)
Supplement: Additional file 1 — Full search strategies. [file 2046-4053-2-109-S1.doc]

Additional file 1: Full search strategies

MEDLINE (through PubMed):

(physical examination OR medical assessment[tiab] OR medical evaluation[tiab] OR physical manifestation*[tiab]) AND (abuse[tiab] OR domestic violence OR battering OR maltreatment OR non-accidental injury) AND (adolescent OR child OR infant OR adolescen*[tiab] OR child*[tiab] OR infan*[tiab] OR newborn*[tiab])

MEDLINE (through OVID):

1 (screen* adj3 (instrument* or examination or evaluation)).tw.

2 (diagnostic adj3 (instrument* or examination or evaluation)).tw.

3 ((medical or physical or diagnostic) adj3 inspection*).tw.

4 ((medical adj5 assessment) or (medical adj5 examination) or (medical adj5 evaluation) or (physical adj5 examination)).ti,ab. or physical examination.mp.

5 1 or 2 or 3 or 4

6 exp child abuse/ or child abuse.mp.

7 ((domestic violence or child abuse or maltreatment or munchhausen or shaken or neglect* or batter* or non-accidental injur*) adj3 (child* or infan* or adolescen* or toddler* or neonat* or newborn* or baby or babies)).tw.

8 domestic violence/ and (adolescent/ or child/ or infant/ or newborn/ or pediatr*.tw. or paediatr*.tw.)

9 or/6-8

10 5 and 9

CINAHL:

S1 (MH "Child Abuse+")

S2 TX child W3 abuse

S3 TX child W3 maltreatment

S4 S1 or S2 or S3

S5 TX medical N5 assessment

S6 TX medical N5 examination

S7 TX medical N5 evaluation

S8 (MH "Physical Examination+")

S9 TX physical N3 examination

S10 S5 or S6 or S7 or S8 or S9

S11 S4 and S10

S12 TX (munchhausen or shaken or neglect* or batter* or non-accidental injur*) W3 (child* or infan* or adolescen* or toddler* or neonat* or newborn* or baby or babies)

S13 S4 or S12

S14 S10 and S13

EMBASE:

1 (screen* adj3 (instrument* or examination or evaluation)).tw.

2 (diagnostic adj3 (instrument* or examination or evaluation)).tw.

3 ((medical or physical or diagnostic) adj3 inspection*).tw.

4 ((medical adj5 assessment) or (medical adj5 examination) or (medical adj5 evaluation) or (physical adj5 examination)).ti,ab. or physical examination.mp.

5 1 or 2 or 3 or 4

6 exp child abuse/ or child abuse.mp.

7 ((domestic violence or child abuse or maltreatment or munchhausen or shaken or neglect* or batter* or non-accidental injur*) adj3 (child* or infan* or adolescen* or toddler* or neonat* or newborn* or baby or babies)).tw.

8 domestic violence/ and (adolecesent/ or child/ or infant/ or newborn/ or pediatr*.tw. or paediatr*.tw.)

9 or/6-8

10 5 and 9

PSYCINFO:

1 (screen* adj3 (instrument* or examination or evaluation)).tw.

2 (diagnostic adj3 (instrument* or examination or evaluation)).tw.

3 ((medical or physical or diagnostic) adj3 inspection*).tw.

4 ((medical adj5 assessment) or (medical adj5 examination) or (medical adj5 evaluation) or (physical adj5 examination)).ti,ab. or physical examination.mp.

5 1 or 2 or 3 or 4

6 exp child abuse/ or exp abuse reporting/ or child abuse reporting/ or child neglect/ or domestic violence/ or failure to thrive/ or exp munchausen syndrome by proxy/ or physical abuse/ or exp violent crime/

7 ((domestic violence or child abuse or maltreatment or munchhausen or shaken or neglect* or batter* or non-accidental injur*) adj3 (child* or infan* or adolescen* or toddler* or neonat* or newborn* or baby or babies)).tw.

8 6 or 7

9 5 and 8

ERIC:

1 (screen* adj3 (instrument* or examination or evaluation)).tw. (849)

2 (diagnostic adj3 (instrument* or examination or evaluation)).tw. (649)

3 ((medical or physical or diagnostic) adj3 inspection*).tw. (12)

4 ((medical adj5 assessment) or (medical adj5 examination) or (medical adj5 evaluation) or (physical adj5 examination)).ti,ab. or physical examination.mp. (1110)

5 ((domestic violence or child abuse or maltreatment or munchhausen or shaken or neglect* or batter* or non-accidental injur*) adj3 (child* or infan* or adolescen* or toddler* or neonat* or newborn* or baby or babies)).tw. (8897)

6 child abuse/ or child neglect/ or ((family problems/ or family violence/ or physical abuse*.tw.) and (child* or infan* or adolescen* or toddler* or neonat* or newborn* or baby or babies)).mp. (10514)

7 1 or 2 or 3 or 4 (2558)

8 5 or 6 (11699)

9 7 and 8 (90)
