# Supplementary material for: Insufficient evidence for the use of a physical examination to detect maltreatment in children without prior suspicion: a systematic review
Source: Syst Rev. 2013 Dec 6;2:109. doi: 10.1186/2046-4053-2-109 (PMC4029283; doi:10.1186/2046-4053-2-109)
Supplement: Additional file 2 — 2 x 2 contingency tables of included studies. [file 2046-4053-2-109-S2.docx]

Additional file 2: 2 x 2 contingency tables of the results of the included studies.

| Author (year) |  | Reference standard confirms child maltreatment | Reference standard does not confirm child maltreatment | Total |
| --- | --- | --- | --- | --- |
| Afifi (2003) [28] | Signs of maltreatment upon physical examination | 75 | 6 | 81 (burns 30, bruises 20, scars 19, scratches 10, bite marks 2) |
|  | No signs of maltreatment upon physical examination | 0 | 474 | 474 |
|  | Total | 75 | 480 | 555 |

| Author (year) |  | Reference standard confirms child maltreatment | Reference standard does not confirm child maltreatment | Total |
| --- | --- | --- | --- | --- |
| Palazzi (2005) [29] | Signs of maltreatment upon physical examination | Skin lesions: 75 Oral lesions: 8 | Skin lesions: 1102  Oral lesions: 115 | Skin lesions: 1177  Oral lesions: 123  Present or past burns, fractures and head trauma are presented separately in the original article. However, it is unclear whether this is assessed during physical examination, therefore these results are not included in this review.  The number of children with at least 1 finding upon physical examination is unknown |
|  | No signs of maltreatment upon physical examination | Skin lesions: 119  Oral lesions: 188 | Skin lesions: 8214  Oral lesions: 8826 | Skin lesions: 8333  Oral lesions: 9014 |
|  | Total | Skin lesions: 194  Oral lesions: 196 | Skin Lesions: 9316  Oral lesions: 8941 | Skin lesions: 9510  Oral lesions: 9137 |

| Author (year) |  | Reference standard confirms child maltreatment | Reference standard does not confirm child maltreatment | Total |
| --- | --- | --- | --- | --- |
| Rosenberg (1982) [30] | Signs of maltreatment upon physical examination | Unkempt: 7  Bruises, burns, human bites: 5  Bald occiput*: 0 | Unkempt: 30  Bruises, burns, human bites: 13  Bald occiput*: 14 | Unkempt: 37  Bruises, burns, human bites: 18  Bald occiput*: 14  The number of children with at least 1 finding upon physical examination is unknown |
|  | No signs of maltreatment upon physical examination | Unkempt: 13  Bruises, burns, human bites: 15  Bald occiput*: 20 | Unkempt: 423  Bruises, burns, human bites: 440  Bald occiput*: 440 | Unkempt: 436  Bruises, burns, human bites: 455  Bald occiput*: 460 |
|  | Total | Unkempt: 20  Bruises, burns, human bites: 20  Bald occiput*: 20 | Unkempt: 453  Bruises, burns, human bites: 453  Bald occiput*: 454 | Unkempt: 473  Bruises, burns, human bites: 473  Bald occiput*: 474 |

*This is no longer considered a sign of child abuse.
